# Resuscitation of Dormant “Non-culturable” *Mycobacterium tuberculosis* Is Characterized by Immediate Transcriptional Burst

**DOI:** 10.3389/fcimb.2019.00272

**Published:** 2019-07-30

**Authors:** Elena G. Salina, Artem S. Grigorov, Oksana S. Bychenko, Yulia V. Skvortsova, Ilgar Z. Mamedov, Tatyana L. Azhikina, Arseny S. Kaprelyants

**Affiliations:** ^1^Bach Institute of Biochemistry, Research Center of Biotechnology of the Russian Academy of Sciences, Moscow, Russia; ^2^Shemyakin and Ovchinnikov Institute of Bioorganic Chemistry, Russian Academy of Sciences, Moscow, Russia

**Keywords:** resuscitation, *M. tuberculosis*, non-culturability, dormancy, transcriptional burst, small non-coding RNAs

## Abstract

Under unfavorable conditions such as host immune responses and environmental stresses, human pathogen *Mycobacterium tuberculosis* may acquire the dormancy phenotype characterized by “non-culturability” and a substantial decrease of metabolic activity and global transcription rates. Here, we found that the transition of *M. tuberculosis* from the dormant “non-culturable” (NC) cells to fully replicating population *in vitro* occurred not earlier than 7 days after the start of the resuscitation process, with predominant resuscitation over this time interval evidenced by shortening apparent generation time up to 2.8 h at the beginning of resuscitation. The early resuscitation phase was characterized by constant, albeit low, incorporation of radioactive uracil, indicating *de novo* transcription immediately after the removal of the stress factor, which resulted in significant changes of the *M. tuberculosis* transcriptional profile already after the first 24 h of resuscitation. This early response included transcriptional upregulation of genes encoding enzymes of fatty acid synthase system type I (FASI) and type II (FASII) responsible for fatty acid/mycolic acid biosynthesis, and regulatory genes, including *whiB6* encoding a redox-sensing transcription factor. The second resuscitation phase took place 4 days after the resuscitation onset, i.e., still before the start of active cell division, and included activation of central metabolism genes encoding NADH dehydrogenases, ATP-synthases, and ribosomal proteins. Our results demonstrate, for the first time, that the resuscitation of dormant NC *M. tuberculosis* is characterized by immediate activation of *de novo* transcription followed by the upregulation of genes controlling key metabolic pathways and then, cell multiplication.

## Introduction

One-third of the human population is estimated to be latently infected with *Mycobacterium tuberculosis*, a causative agent of tuberculosis (TB), without overt disease symptoms (World Health Organization, 2018). Being under immune control of the host, latent TB infection presents a constant risk of disease reactivation, which constitutes 10% over a lifetime in the general population and 10% per year in immunocompromised patients (Pawlowski et al., 2012; O'Garra et al., 2013; Scriba et al., 2016; Veatch and Kaushal, 2018). The clinical status of latent TB is traditionally associated with the transition of *M. tuberculosis* to a dormant state in response to non-optimal growth conditions *in vivo* due to activation of the host immune response (Zhang, 2004). Dormancy is a specific physiological state characterized by significant cessation of metabolic activity and growth, whereas resuscitation from dormancy is a process of restoring cell activity followed by bacterial multiplication, which in case of *M. tuberculosis* can lead to disease progression. Therefore, understanding the mechanisms underlying *M. tuberculosis* resuscitation may provide clues to the development of control measures in order to inhibit reactivation of latent infection, reduce disease severity in infected patients, and prevent pathogen transmission in the population.

Although there are several *in vivo* models of *M. tuberculosis* latency and reactivation, including mice (McCune and Tompsett, 1956; Scanga et al., 1999; Radaeva et al., 2005), guinea pigs (Ordway et al., 2010), and rabbits (Manabe et al., 2008; Subbian et al., 2012), they do not reproduce disease pathology and immune control observed in humans. The cellular composition of murine TB granulomas is similar to that of human granulomas, with the exception of the absence of multinucleated giant cells, however, mouse TB lesions lack tissue necrosis, which is the pathological hallmark of human TB granulomas (Dutta and Karakousis, 2014). Granulomas in guinea pigs and rabbits more closely approximate the human granuloma; while TB granulomas in standard mouse models are not hypoxic, tissue hypoxia is observed in those of guinea pigs and rabbits (Dutta and Karakousis, 2014). TB models in non-human primates most closely resemble human disease (Kaushal et al., 2012; Peña and Ho, 2015, 2016), however, they are expensive and time-consuming. Furthermore, the existing *in vivo* TB reactivation models are aimed to study host immune response after the reactivation of infection rather than the mechanisms of bacterial resuscitation (Mehra et al., 2011; Foreman et al., 2016).

*In vitro* modeling is a cost-effective strategy, which enables investigation of *M. tuberculosis* resuscitation process in detail and identify principal molecular players, providing necessary clues for further *in vivo* experiments (Veatch and Kaushal, 2018). However, despite extensive attempts for modeling of *M. tuberculosis* dormancy *in vitro*, including the well-known Wayne model of non-replicating state under hypoxic conditions (Wayne, 1976; Wayne and Hayes, 1996), dormant bacilli obtained in the majority of *in vitro* models were fully culturable, whereas bacteria isolated from *in vivo* models of latent TB are “non-culturable” (NC) (Dhillon et al., 2004; Biketov et al., 2007). “Non-culturability” is a specific term for cells that are temporary unable to grow on standard solid media and become culturable only after resuscitation (Oliver, 2005). Therefore, *in vitro* models of *M. tuberculosis* dormancy should reproduce the phenomenon of “non-culturability” and more adequately imitate latent TB in humans and animals. Recently, we have developed an *in vitro* model of *M. tuberculosis* dormancy in K^+^-limiting conditions, in which the bacteria acquired the NC phenotype, high tolerance to rifampicin and isoniazid, and significant (up to 1 × 10^7^ cells/ml) recovery potential (Salina E. et al., 2014; Ignatov et al., 2015). Using the model of dormancy under K^+^ deficiency, we found that the adaptation of *M. tuberculosis* to the NC state was characterized by global transcriptional repression and maintenance of a pool of stable/stored transcripts in dormant cells (Ignatov et al., 2015).

Still, little is known about the resuscitation of dormant *M. tuberculosis* and the mechanisms underlying the transition from the NC to multiplication state. Most studies on *M. tuberculosis* resuscitation modeled reactivation by re-aeration of dormant cells obtained under hypoxia, thus studying only one stress parameter—oxygen deficiency (Du et al., 2016; Iona et al., 2016). Transcription analysis revealed that re-aeration affected multiple metabolic pathways, including downregulation of persistence-associated regulons and upregulation of pathways involved in DNA repair and recombination and synthesis of major cell wall components (Du et al., 2016; Iona et al., 2016), indicating physiological transformation of *M. tuberculosis* in preparation for cell division.

In our previous study, we examined transcriptomic changes in NC *M. tuberculosis* during resuscitation after potassium re-introduction using microarray technology, which revealed a lag phase in the transcriptional initiation of resuscitating cells that lasted for at least 4 days (Salina E. G. et al., 2014). However, according to the level of radioactive uracil incorporation, transcriptional activity was triggered in resuscitating *M. tuberculosis* immediately after switching to growth-favoring conditions. It can be speculated that the failure to detect activation of gene expression at the early resuscitation phase of dormant NC bacilli characterized by global transcriptional repression (Ignatov et al., 2015) can be attributed to the limited sensitivity of the microarray assay (Shendure, 2008). Therefore, in this study, we examined early transcriptional response of *M. tuberculosis* to resuscitation using a more sensitive RNA-seq approach.

Here, we report, for the first time, that the resuscitation of NC *M. tuberculosis* from dormancy involves two-stage transcriptional activation, including immediate *de novo* mRNA synthesis, that precedes cell multiplication. This scenario involves activation of lipid metabolism and cell defense mechanisms followed by the induction of central metabolic reactions.

## Materials and Methods

### Bacteria and Media

Dormant NC *M. tuberculosis* was obtained as described previously (Ignatov et al., 2015). Briefly, *M. tuberculosis* strain H37Rv was initially grown from frozen stocks for 10 days in Sauton medium containing (per liter): 0.5 g KH_2_PO_4_, 1.4 g MgSO_4_ · 7H_2_O, 4 g L-asparagine, 60 ml glycerol, 0.05 g ferric ammonium citrate, 2 g sodium citrate, 0.1 ml 1% ZnSO_4_, pH 7.0 (adjusted with 1 M NaOH) and supplemented with ADC and 0.05% Tween 80, at 37°C with agitation (200 rpm). The starter culture was inoculated into fresh medium (same composition) and incubated for another 10 days with agitation (200 rpm) until its optical density at 600 nm (OD600) reached 4.0. These bacteria were then inoculated (5 × 10^5^ cells/ml) into K+-deficient Sauton medium (containing 8.9 g Na_2_HPO_4_ · 12 H_2_O instead of 0.5 g KH_2_PO_4_) and grown at 37°C, with agitation (200 rpm). After 14–15 days of culture, when CFU started to decrease, rifampicin (5 μg/ml) was added to eliminate culturable bacteria and to obtain the NC population with the “zero-CFU” phenotype.

### Resuscitation of “Non-culturable” Cells

Dormant NC cells were harvested by centrifugation (20 min at 5,000 rpm), washed twice with fresh Sauton media and diluted 5 times from initial culture volume with “resuscitation media” which is standard Sauton medium containing 0.6% glycerol (Shleeva et al., 2011) with ADC and Tween-80 (0.05% v/v), and supplemented with an equal volume of used culture supernatant, prepared as previously described. Bacterial cultures were incubated with agitation (150 rpm) at 37°C and harvested at appropriate time points for colony forming units (CFU) and most probable numbers (MPN) counting, radioactive uracil incorporation, DPI-reductase activity measurement and isolation of RNA. In some cases, resuscitation procedure was performed in the presence of 5 μg/ml of rifampicin.

### Cell Viability Estimation

To assess cell viability, 10-fold serial dilutions of *M. tuberculosis* cultures were plated in triplicate onto solidified Sauton agar supplemented with ADC and incubated at 37°C for 25 days, after which CFUs were counted. To assess the proportion of bacteria with the ability to resuscitate in liquid medium by MPN assay, 10-fold bacterial dilutions were resuspended in ADC-supplemented Sauton medium diluted 1:1 (v/v; final glycerol concentration, 0.6%) and seeded into 48-well Corning microplates, which were incubated statically at 37°C for 30 days. The wells with visible bacterial growth were counted as positive, and MPN values were calculated using standard statistical methods (de Man, 1975).

### Incorporation of Radioactively Labeled Uracil

One μl of 5,6-3H uracil (1 mCi) was added to 1 ml culture samples and incubated at 37°C with agitation for 20 h. Two hundred microliters of this culture was placed in 3 ml 7% ice-cold CCl_3_COOH and incubated at 0°C for 20 min, followed by filtration through a glass microfiber filter (Whatman). Precipitated cells were washed with 3 ml 7% CCl_3_COOH and 6 ml 96% ethanol. Filters were placed in 10 ml scintillation mixture; impulse counts were determined by LS analyzer (Beckman Instruments) and expressed as counts per minute (cpm).

### DPI-Reductase Activity Measurement

The activity of the initial part of respiratory chain (Complex I) of cells was evaluated by estimation of their activity to reduce the artificial electron acceptor−2,6-dichlorophenol-indophenol (DPI) in the presence of menadione by measurement of color changes in the optical density at 600 nm. The reaction mixture (1 ml) contained: 0.5 mM 2,6-DPI; 0.145 mM menadione and *M. tuberculosis* cell suspension (1 × 10^7^ cells) in 0.1 M phosphate buffer pH 7.0 at 37°C. The activity was calculated as pmols of DPI on 1 ml of cell suspension per minute, defining it as the average of the results of 3 measurements, while the relative error did not exceed 5%.

### Isolation of RNA

Bacterial cultures were rapidly cooled on ice, centrifuged, and total RNA was isolated by phenol-chloroform extraction after cell disruption with BeadBeater (BioSpec Products) as previously described (Rustad et al., 2009). After isolation, RNA was treated with Turbo DNase (Life Technologies) to remove traces of genomic DNA, and purified with the RNeasy mini kit (Qiagen). Amounts and purity of RNA were determined spectrophotometrically; integrity of RNA was assessed in 1% agarose gel. Semi-quantitative evaluation of rRNA in total RNA samples was performed by Gel-Pro Analyzer software (Meyers Instruments).

### Illumina Sequencing

RNA samples were depleted of 16S and 23S rRNA using Ribo-Zero rRNA Removal Kit for Gram-Positive Bacteria (Epicenter). Sequencing libraries were generated using the resulting ribosomal transcript-depleted RNA and the TruSeq Stranded mRNA Library Prep Kit (Illumina) (Run 1) or NEBNext® Ultra™ II Directional RNA Library Prep Kit for Illumina (Run 2) according to the manufacturers' protocol. Sequencing was performed using the Illumina HiSeq 4000 (Run 1) or HiSeq 2500 (Run 2). Experiments were performed in triplicates.

### Processing of RNA-seq Data

After quality control evaluation and trimming of bad qualitative reads the reads were mapped on the reference *M. tuberculosis* genome (AL123456.3, http://www.ncbi.nlm.nih.gov/) by Bowtie2 (Langmead and Salzberg, 2012). The alignment was performed with the “–local” option, which allows leaving 5′ and 3′ ends uncharted. Calculation of the mapped reads for all genes was performed using functions of the Feature Counts package (Liao et al., 2014) built into the author's script. Gene expression was represented in form of reads per kilobase per million (RPKM), for the calculation of which only unambiguously mapping reads were used. Differentially expressed genes were identified by the software package DESeq2 (Love et al., 2014). The genes were considered to be differentially expressed, if the *p*-adjusted value was <0.1, and the expression change module (FC, Fold change) was not <3. Further distribution of genes according functional categories was performed using the Mycobrowser database (https://mycobrowser.epfl.ch/).

### Quantitative Real-Time PCR

One microgram of total RNA was used for cDNA synthesis with random hexanucleotides and SuperScript III reverse transcriptase (Life Technologies). Quantitative PCR was performed using qPCRmix-HS SYBR (Evrogen) and the Light Cycler 480 real-time PCR system (Roche); cycling conditions were as follows: 95°C for 20 s, 60°C for 20 s, 72°C for 30 s, repeat 40 times; primers are listed in Table S1. In the end of amplification, a dissociation curve was plotted to confirm specificity of the product. All real-time experiments were repeated in triplicate. The results were normalized against the 16S rRNA gene.

## Results

### Dynamics of NC *M. tuberculosis* Resuscitation

**Figure 1:**
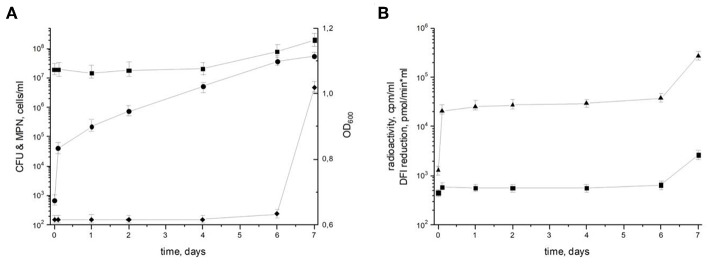
Resuscitation of dormant non-culturable *M. tuberculosis* bacilli. Changes in cell characteristics during the transition from NC to actively growing state. **(A)** Most probable number (squares) MPN/ml; colony forming units (circles) CFU/ml; optical density (diamonds) OD_600_. **(B)** Radioactive incorporation of uracil (triangles) cpm/ml; DPI reduction (squares) pmol/(min × ml). This experiment was repeated five times with similar results.

To characterize the resuscitation phenomenon and the transition of dormant *M. tuberculosis* from the NC state (“zero-CFU” phenotype) to the multiplication phase, dormant NC bacilli were washed and inoculated into fresh resuscitation medium. Although there were no changes in the optical density (OD) 600 or most probable number (MPN) corresponding to the maximum count of potentially viably cells in the population until day 7 (the time point when cell multiplication begins), the number of culturable bacteria estimated by colony forming units (CFU) increased by about 10 times immediately after washing and continued to rise, reaching 5.7 × 10^7^ CFU/ml at day 7 (Figure 1). Estimation of the apparent generation time revealed its gradual increase from 2.8 h (from NC state to day 1 of resuscitation) to 16 h (from day 6 to 7 of resuscitation). As the generation time for replicating *M. tuberculosis* in this medium is 18–20 h, its shortening indicates the prevalence of resuscitation over multiplication during transition of NC *M. tuberculosis* to replicating state. In addition, the level of metabolic activity assessed by incorporation of radioactively labeled uracil and DPI-reductase activity, which reflects functioning of the initial part of the respiratory chain, was constantly low until a steep increase at day 7, which also supports the predominance of resuscitation over cell division up to day 6 (Figure 1). The constant number of potentially viable cells both in NC state and resuscitating mycobacteria estimated by MPN assay also supports the notion of resuscitation preceding multiplication.

### Ribosomal RNA Status

**Figure 2:**
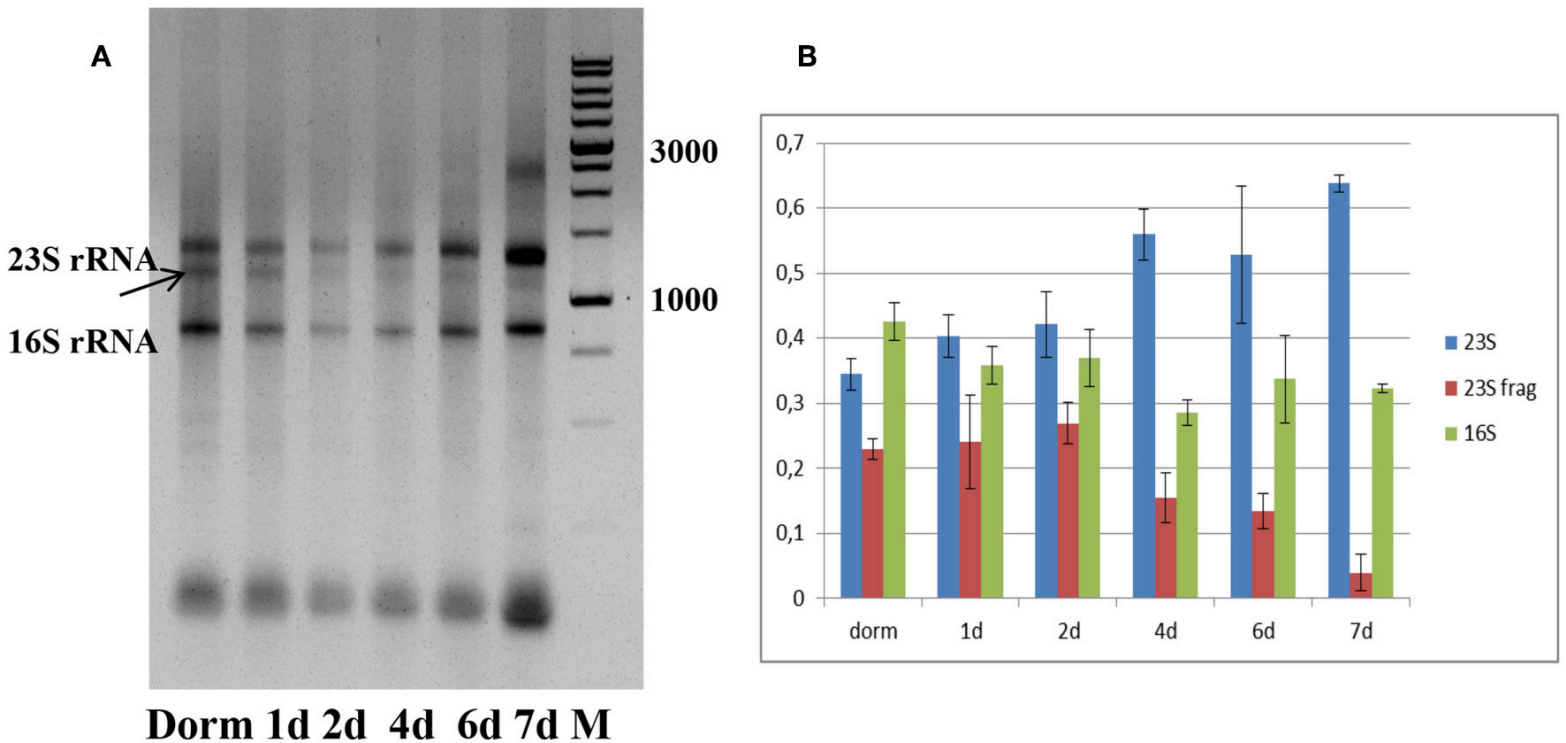
Ribosomal RNA status during resuscitation. **(A)** 1.0% agarose gel profiling of total RNA samples. Arrow indicates the 23S rRNA fragment characteristic for *M. tuberculosis* NC state. M−1 kb DNA ladder (SibEnzyme, Russia). **(B)** Changes in the proportion of 23S/23S fragment/16S rRNAs in the process of resuscitation. The proportions obtained by image 2A processing by Gel-Pro Analyzer software. The results of three independent RNA isolation experiments were presented.

Total RNA profiling in dormant NC *M. tuberculosis* (Dorm) revealed the presence of an additional fragment below the 23S rRNA band (Figure 2A), which was likely a product of specific 23S rRNA cleavage characteristic for NC *M. tuberculosis* (Ignatov et al., 2015). This 23S rRNA fragment was detected over the whole resuscitation period—from dormancy to day 7 (Figure 2A).

We estimated the relative ratio of 23S, 23S fragment and 16S rRNAs at each time point of resuscitation of bacterial culture (Figure 2B), and found that it was very similar in dormancy and at days 1 and 2. From day 4 we noticed the increase of intact 23S rRNA amount and corresponding decrease of 23S fragment amount. This tendency is most pronounced at day7, where integrity of 23S rRNA is the highest.

### RNA-seq Analysis

**Figure 3:**
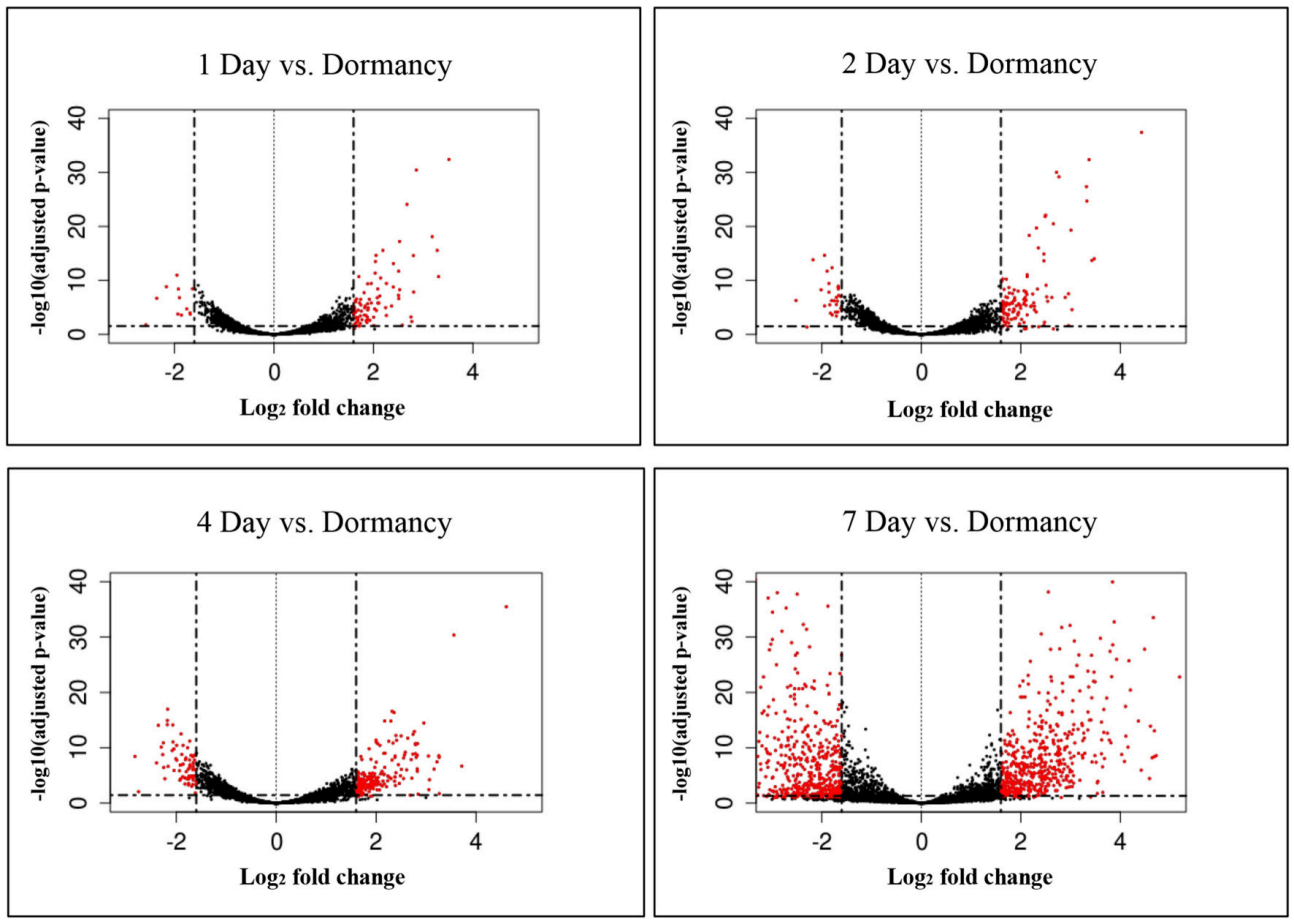
Volcano plots of differentially expressed genes. Fold changes between genes were plotted. Criteria of >3 x change (log2FC > 1.6) in expression and <0.1 p-adj-value were used to define significantly changed genes. Differentially expressed genes which met the criteria are shown in red, and those which were expressed at lower levels are shown in black.

**Figure 4:**
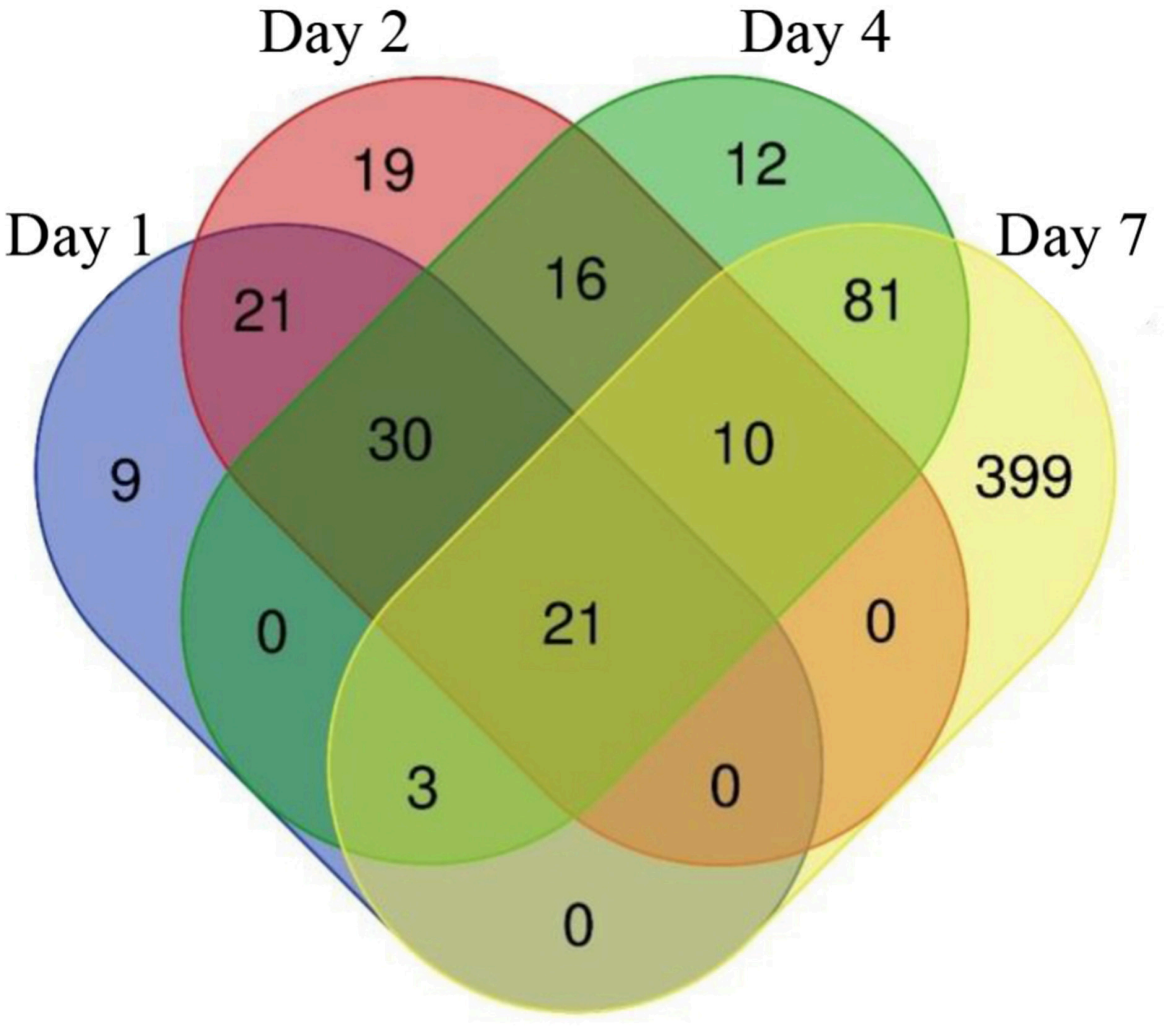
Venn diagram showing the number of differentially expressed genes in days 1, 2, 4, and 7 comparing to dormant NC state.

The constant, albeit low incorporation of radioactive uracil indicative of *de novo* transcription in resuscitating cells immediately after the resuscitation start (Figure 1) prompted us to perform dynamic transcriptome profiling over the resuscitation period by RNA-seq at 5 time points: dormant NC state, days 1, 2, 4, and 7. Using the software package DESeq2, we identified genes differentially expressed in resuscitating compared to dormant *M. tuberculosis* (Table S2). The results indicated that the total number of differentially expressed genes gradually increased over time, reaching maximum at day 7. Volcano plots clearly demonstrate that according to the number of upregulated transcribed genes and transcription intensity (log_2_FC ≥ 1.6), the resuscitation process could be divided into two phases: before and after day 4 (Figure 3). The Venn diagram shows the number of differentially expressed protein-coding genes (log_2_FC ≥ 1.6) on different time points over 7-day resuscitation and their intersections revealed that 21 genes were expressed throughout the whole period, 21were common for days 1 and 2, 45 started expression at day 2, 93—at day 4, and 399—at day 7 (Figure 4).

The functional analysis of differentially expressed genes was performed using the Mycobrowser database and the results for genes with expression fold change exceeding 3 (log_2_FC ≥ 1.6) are shown in Table S2.

### Transcriptional Changes at the Early Resuscitation Phase

Comparison of RPKM values for individual genes at days 1, 2, 4, and 7 with those at the NC state and expression analysis revealed two groups of genes differing in transcription dynamics (Table S2): the first contained genes activated at days 1 and 2 and possibly downregulated later, and the second—those activated after day 4.

**Figure 5:**
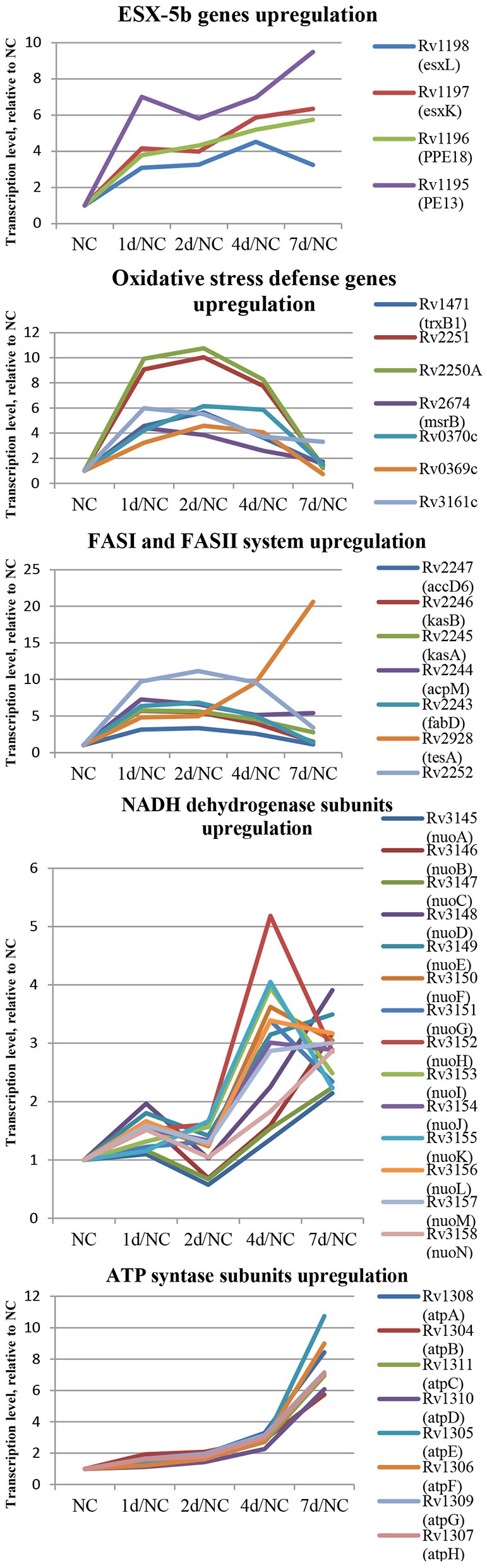
Dynamics of several functional groups expression during resuscitation. Differential expression is given relative to dormant NC state.

In the first group, 84 protein-coding genes showed more then 3-fold upregulation at day 1. The prominent increase was observed for genes encoding enzymes of fatty acid synthase system type I (FASI) and type II (FASII) (Figure 5) responsible for biosynthesis of fatty/mycolic acids (Singh et al., 2011), including diacylglycerol kinase (Rv2252), meromycolate extension acyl carrier protein (Rv2244, *acpM*), holo acyl-carrier protein synthase (Rv2523c, *acpS*), malonyl CoA-acyl carrier protein transacylase (Rv0649, *fabD*), keto-acyl-carrier protein synthases (Rv2245, *kasA* and Rv2246, *kasB*), atty acid synthase (Rv2524c, *fas*), acetyl/propionyl-CoA carboxylase (Rv2247, *accD6*), thioesterase (Rv2928, *tesA*), polyketide synthase associated proteins (Rv1528c, *papA4*), and enoyl-CoA hydratase (Rv1472, *echA12*).

The genes encoding redox-sensing transcription factor WhiB6 and transcriptional regulator Rv3830c belonging to the TetR-family were also considerably induced after 24 h of resuscitation. Similar expression shifts were observed for the *pe13* (Rv1195), *ppe18* (Rv1196), *esxK* (Rv1197), and *esxL* (Rv1198) genes, coding the components of the ESX-5b secretion system (Shah and Briken, 2016).

Several genes of mycobacterial defense systems, such as *hsp* (Rv0251c) encoding a molecular chaperone heat shock protein, *clpB* (Rv0384c) encoding endopeptidase which removes oxidized proteins, and antitoxin *vapB12* (Rv1721c) encoding antitoxin, were upregulated at the resuscitation start. A similar activation pattern was observed for *trxB1*(Rv1471) coding for thioredoxin, which participates in various redox reactions through reversible oxidation of dithiol, and *msrB* (Rv2674) coding for peptide methionine sulphoxide reductase, a repair enzyme for proteins inactivated by oxidation. In addition, the expression of genes encoding some enzymes involved in redox reactions, including dioxygenase Rv3161c, oxidoreductases Rv3352c, Rv0369c, and Rv0370c as well as electron acceptors flavoproteins Rv2250A and Rv2251 was also upregulated, indicating intensification of redox processes in the early resuscitation phase. The *pyrE* (Rv0382c) gene for orotate phosphoribosyl transferase involved in pyrimidine biosynthesis was also significantly activated.

The transcriptional profile of *M. tuberculosis* at resuscitation day 2 closely resembled that at day 1, however, more genes (107) were upregulated by >3-fold. In particular, the transcription activity of the majority of genes listed above, namely, those belonging to FAS I and FAS II, fluctuated at the same level or tended to be slightly higher than at 24 h. Transcription of *whiB6* and *hsp* tended to increase further at day 2.

### Transcriptional Changes at the Late Resuscitation Phase

In the second group, the spectrum of genes activated at the late phase of resuscitation (days 4–7) was significantly different from that in the first group. Among the late-response genes, there were those related to central metabolism pathways, such as NADH dehydrogenase subunits *nuoEFGHIJKLM* (Rv3149-Rv3157). Activation of *qcrA* (Rv2195, iron-sulfur protein), *qcrB* (Rv2196), and *qcrC* (Rv2197, ubiquinol-cytochrome C reductase), and *ctaE* (Rv2193, cytochrome C oxidase) indicated initiation of aerobic respiration at day 4. The *atpA-G* (Rv1304-Rv1311) genes coding for the ATPase complex were also upregulated. Activation of enzymes belonging to the tricarboxylic acid cycle: *sdhAB* (Rv3318, Rv3319, succinate dehydrogenase subunit), *acn* (Rv1475c, aconitase)*, sucCD* (Rv0951, Rv0952, succinyl-CoA synthetase subunits), *gltA2* (Rv0896, citrate synthase I), *ndkA* (Rv2245c, nucleoside phosphate kinase participating in nucleoside biosynthesis), and *pntAb* and *pntB* (Rv0156, Rv0157, transhydrogenase subunits providing transhydrogenation between NADH and NADP) clearly indicated initiation of energy metabolism after resuscitation day 4.

The expression of genes coding for the enzymes of the tryptophan biosynthesis pathway (*trpABCS*, Rv1611-Rv1613, and Rv3336c, respectively) and proteins of the antigen 85 complex [antigen 85 and cord-factor, *fbpA (*Rv3804c) and *fbpD* (Rv3803c), respectively] was also increased. The upregulation of *rmlBC* (Rv3464-Rv3465, rhamnose biosynthesis) and *ppiA* (Rv0009, peptidyl-prolyl cis-trans isomerase accelerating protein folding) indicated the start of central metabolic processes, whereas that of *ahpCD* (Rv2428-Rv2429, involved in oxidative stress response) suggested unbalanced metabolism at this stage of resuscitation.

To confirm the RNA-seq data, we assessed the expression dynamics of selected genes by qPCR using the 16S rRNA gene as a reference. During resuscitation, the correlation between the RNA-seq (RPKM) and qPCR data was moderate to high as evidenced by Spearman correlation coefficient from 0.60 to 0.95 (Table S3).

Overall, these results reveal that transcriptional changes in resuscitating *M. tuberculosis* may occur in two steps: at first, activation of fatty acid biosynthesis, defense systems, and transcriptional regulators is provided, and then, the induction of central metabolism and respiration takes places prior to cell multiplication (Figure 5).

### Non-coding Transcriptome

Recent studies have reported upregulation of non-coding (nc) RNAs in *M. tuberculosis* under different stress conditions (Arnvig and Young, 2012; Ignatov et al., 2015; Del Portillo et al., 2018). Thus, we have previously found that MTS0997, MTS1338, and MTS2823 were the most abundant ncRNAs in dormant NC mycobacteria subjected to potassium deficiency (Ignatov et al., 2015). During resuscitation, only one ncRNA, MrsI (MTB000142, ncRv11846), was upregulated: 4-fold at day 1 and 6- to 7-fold at days 2 and 4 (Table S2). MTS2823 (MTB000078) and MTS0997 (mcr11, MTB000063) were downregulated 4-fold at day 1 and this level remained constant, whereas MTS1338 expression changes were not statistically significant.

**Figure 6:**
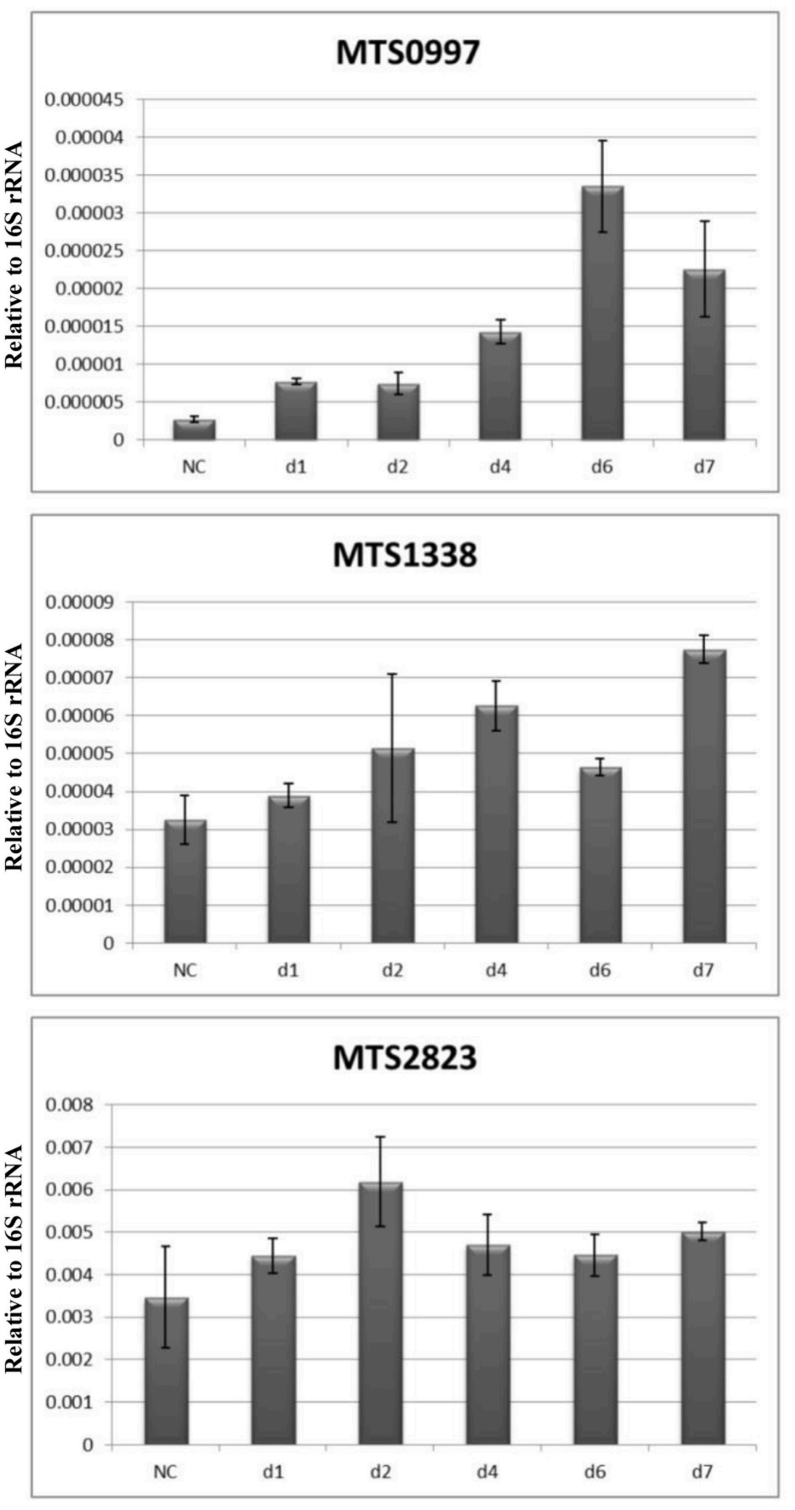
Dynamics of ncRNAs expression during resuscitation. qPCR data are normalized to 16S rRNA transcription level.

These data were further validated by qPCR at the NC stage and at days 1, 2, 4, and 7. The results revealed that the expression of MST2823 was unchanged, that of MTS1338 was slightly upregulated at day 4 and increased by 2- to 3-fold at day 7, and that of MTS0997 also increased by about 10-fold at day 7 (Figure 6).

### Confirmation of the *de novo* Transcription at the Early Resuscitation Phase

To confirm the early transcriptional response and *de novo* mRNA synthesis at resuscitation, we treated resuscitating *M. tuberculosis* by rifampicin, a blocker of RNA-polymerase activity. The results revealed no changes in the OD600 and metabolic activity until day 7, and even a log reduction in CFU by day 2, indicating that transcriptional blockade inhibited resuscitation (Figure S1).

The expression of seven most upregulated genes representing the early transcriptional response was examined by qPCR at days 1 and 2 in resuscitating *M. tuberculosis* treated or not with rifampicin and compared to that in NC bacteria. The following genes were analyzed: Rv2243 (*fabD*), Rv2246 (*kasB*), Rv3160, Rv0251c (*hsp*), Rv0384 (*clpB*), Rv2674 (*msrB*), and Rv1471 (*trxB1*). Changes observed in the transcription of these genes at resuscitation days 1 and 2 were abolished by rifampicin treatment (Figure S2A), confirming the *de novo* mRNA synthesis at the early stages of *M. tuberculosis* resuscitation.

Finally, we performed qPCR analysis of ncRNAs and found that rifampicin caused no difference in the transcription rate of ncRNAs MTS2823 and MTS1338 (Figure S2B), indicating that these ncRNAs are stable and not being newly synthesized during resuscitation. However, the upregulation of MTS0997 and MrsI observed at days 1 and 2 was completely blocked by rifampicin, indicating that their *de novo* synthesis is required at the early resuscitation stage of *M. tuberculosis*.

## Discussion

In the present study, we examined the resuscitation of dormant *M. tuberculosis* driven to the NC phenotype by K^+^ deficiency. K^+^ is crucial for the maintenance of an electrochemical gradient and proton motive force in the membrane and for the regulation of intracellular pH and osmotic pressure in both eukaryotic and bacterial cells (Epstein, 2003). In *M. tuberculosis*, low K^+^ concentrations result in the inability to maintain acceptable intracellular pH levels in mildly acidic conditions (Sturgill-Koszycki et al., 1994), thus decreasing cell viability (Rao et al., 2008).

Transcriptional response in several *in vivo* studies (Karakousis et al., 2004; Rachman and Kaufmann, 2007; Garton et al., 2008) may point out potassium deficiency for *M. tuberculosis*. Therefore, we suggested that low K^+^ concentration may be considered as a trigger for development dormant phenotype *in vivo*. We proved experimentally, that K^+^ deficiency *in vitro* induced a dormant phenotype which is characterized by (i) significant decrease in metabolic activity, (ii) tolerance to antibiotics, (iii) changed morphology, (iv) “non-culturability” (Salina E. G. et al., 2014). NC mycobacteria obtained in the K^+^ deficiency model are characterized by a high recovery potential and could be resuscitated in Sauton medium diluted 1:1 with culture supernatant (Figure 1). According to the MPN assay, nearly 50% of NC *M. tuberculosis* population could be driven to the culturable state (Ignatov et al., 2015). According to Figure 1, resuscitation of dormant cells in liquid medium takes at least 6 days. Evidently, that during this period both resuscitation and further cell replication can take place. However, significant shortening of apparent generation time, especially in the very beginning of the reactivation process, steep increase in metabolic activity (radioactive uracil incorporation and DPI reduction) at the same period followed by a plateau, and constant numbers of potentially viable cells (MPN assay) rather indicate the significant prevalence of resuscitation over replication at the first 48 h after the removal of the stress factor. However, the impact of cell multiplication on the overall process cannot be excluded, with increased proportion of replication to the end of resuscitation period.

Earlier, we found that the transition of *M. tuberculosis* to the NC state was accompanied by significant global downregulation of transcriptional activity: by at least 30-fold compared to replicating cells (Ignatov et al., 2015). In this study, we identified, for the first time, the phenomenon of “transcriptional burst” characterized by sharp transcriptional activation of certain genes and significant increase (by 10- to 20-fold) of *de novo* mRNA synthesis at the first 24 h, i.e., time corresponding to one generation (Figure 5 and Table S3), which is consistent with global transcriptional repression during transition to dormancy (Ignatov et al., 2015). The sensitivity of transcriptional activation to rifampicin indicates participation of RNA polymerase, which is fairly stable and present in cells dormant for long periods of time (Trutneva, personal communication). However, the mechanisms underlying the early transcriptional activation in resuscitating *M. tuberculosis* are unclear and require further investigation.

**Figure 7:**
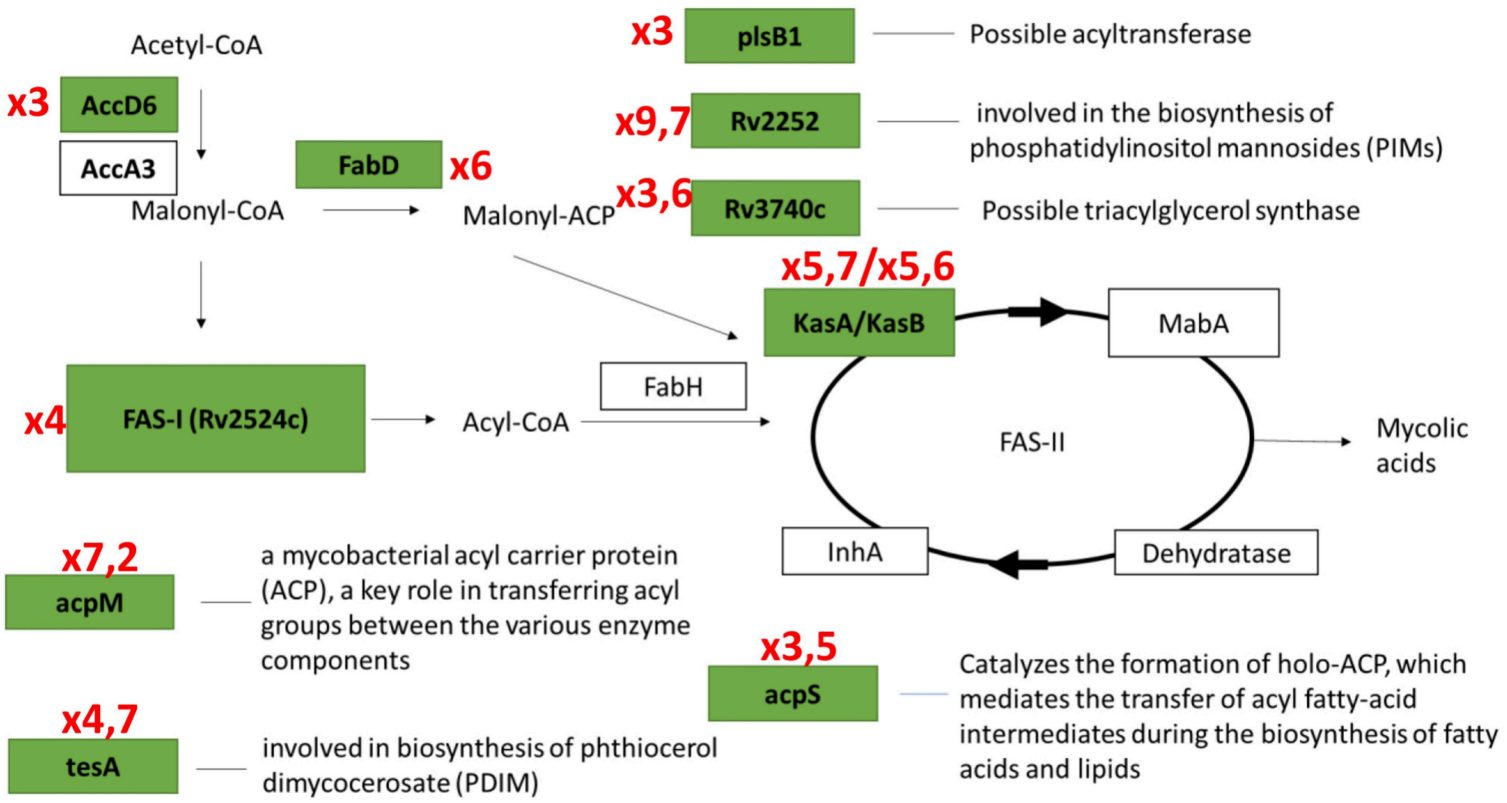
Transcriptional activation of genes involved in fatty acids biosynthesis systems (FASI/FASII) in the early resuscitation phase. Components upregulated at Day1 are given in green boxes. Red numbers designate transcriptional fold changes comparing to dormant NC state.

Results of transcriptome analysis by RNA-seq revealed two groups of genes activated during resuscitation of NC *M. tuberculosis*. In the first group, transcriptional upregulation occurred immediately after stress removal (day 1), suggesting that these genes participate in the early processes necessary for resuscitation rather than multiplication. Interestingly, the majority of genes in fatty acid synthase (FASI and FASII) systems belong to this early activated group (Figure 7). Mycolic acids (2-alkyl, 3-hydroxy long-chain fatty acids) are the major component of the mycobacterial cell wall, constituting about 50% of the dry weight (Barry, 2001), and are crucial for survival and pathogenesis of *M. tuberculosis*, providing resistance to antibiotics and dehydration (Takayama et al., 2005). Our previous results indicate that unlike dormant *M. tuberculosis* isolated from sputum (Garton et al., 2008), NC mycobacteria obtained under K^+^ deficiency have lower lipid content than replicating cells (Salina et al., 2010), suggesting that the activation of fatty acids biosynthesis during resuscitation may compensate the lack of lipids in dormancy providing augmentation of cell wall components for further bacilli multiplication.

We also observed transcriptional induction of heat-shock protein Hsp, a molecular chaperone, and endopeptidase ClpB involved in degradation of misfolded and oxidized proteins and protein turnover. Clp proteases have been identified as factors specific to *M. tuberculosis* reactivation (Sherrid et al., 2010; McGillivray et al., 2015; Du et al., 2016) and mycobacteria lacking ClpB were found to be less fit to recover from the stationary phase or antibiotic exposure *in vitro* and to have lower virulence in mice than the wild type (Vaubourgeix et al., 2015).

WhiB6 belonging to the WhiB superfamily of small global transcriptional regulators is known to control aerobic and anaerobic metabolism, cell division, and virulence of *M. tuberculosis* (Chen et al., 2016). Transcriptional regulators Rv3830c and Rv3160c of the TetR protein family are involved in the regulation of multidrug efflux pumps, response to osmotic stress and toxic chemicals, control of catabolic pathways and differentiation processes, and antibiotic resistance of pathogenic bacteria, including *M. tuberculosis* (Ramos et al., 2005). Therefore, the upregulation of these genes during reactivation from dormancy may reflect metabolic adjustment in resuscitating cells, in particular, neutralization of toxic component produced in catabolic reactions.

We paid special attention to small ncRNAs known to control transcription and translation in bacteria. Typically, ncRNAs are expressed in response to external factors, enabling bacterial adaptation to changing environmental conditions and regulating the key stages of pathogenesis. In our study, significant increase was observed only for MrsI, whose *de novo* (rifampicin-sensitive) synthesis was steady up to the cell multiplication phase at day 7. This ncRNA was recently characterized as a mediator of iron-sparing response but is also activated by other macrophage-related stress factors, and it is suggested that MrsI has an anticipatory function, preparing mycobacteria to potentially unfavorable conditions (Gerrick et al., 2018).

Another ncRNA, MTS0997, was upregulated to a much less extent; however, its *de novo* synthesis was also confirmed. MTS0997 was shown to be involved in the control of *M. tuberculosis* lipid metabolism and was overexpressed under hypoxia in the presence of fatty acids (Aguilar-Ayala et al., 2017; Del Portillo et al., 2018). Given sharp upregulation of FASI/FASII fatty acids synthesis systems at the very start of resuscitation, the increase in MTS0997 expression suggests its involvement in the induction of lipid metabolism in mycobacteria after dormancy.

Two other ncRNAs, MTS1338 and MTS2823, were shown to be significantly represented in the NC *M. tuberculosis* transcriptome. MTS1338 is highly expressed during the stationary growth phase (Arnvig et al., 2011) and at dormancy (Ignatov et al., 2015) and was shown to be a part of the DosR regulon activated in hypoxia (Moores et al., 2017), suggesting its role in the maintenance of *M. tuberculosis* survivability in unfavorable conditions.

MTS2823 belonging to 6S ncRNAs is the most abundant ncRNA in the stationary phase of *M. tuberculosis* (Arnvig et al., 2011). Its *M. smegmatis* homolog Ms1 influences the RNApolymerase level and is suggested to sequester the RNA polymerase core in a cache of inactive enzymes, which can be reactivated when needed (Sikova et al., 2019). Such a mechanism could be critical when the demand for RNA polymerase activity increases after environmental changes such as nutrient availability and outgrowth from the stationary phase. Our quantitative analysis demonstrated constant levels of MTS2823 and its very slight increase of MTS1338 only after resuscitation day 4, which should be mainly due to high ncRNA stability (Ignatov et al., 2015; Moores et al., 2017), rather than *de novo* synthesis. Therefore, we can conclude that MTS1338 and MTS2823 are not involved in the resuscitation process after K^+^-limiting conditions but are preserved through the NC state as stable transcripts accumulated at the time of active *M. tuberculosis* growth.

After 4 days of resuscitation, a group of “late” genes, including those regulating central metabolism (respiration, ATP-synthesis, TCA cycle activity, and translation), are activated, suggesting their role in the initiation of cell division. It is interesting that the five genes coding for resuscitation-promoting factors (Rpfs) did not show significant activation before the start of cell division; only *rpfE* was significantly upregulated at day 7, which coincides with the multiplication onset. In our previous study, we found that only *rpfB* was induced after 8 days of resuscitation from the NC state (Salina E. G. et al., 2014). These findings are consistent with late activation of Rpfs during resuscitation of *M. smegmatis* (Shleeva et al., 2013).

In conclusion, the application of RNA-seq in combination with qPCR enabled us to detect transcriptional changes and *de novo* mRNA synthesis at the early stage of *M. tuberculosis* resuscitation from the dormant NC state, which preceded the activation of genes controlling central metabolism. Our findings revealed, for the first time, the occurrence of an immediate transcriptional burst at the very start of pathogen resuscitation, which is followed by two-phase changes in the expression profile and then, cell multiplication. The described phenomenon of transcriptional activation at the early stage of *M. tuberculosis* recovery from dormancy warrants further investigation to provide comprehensive understanding of the mechanisms underlying pathogen transition from dormancy to replication, which is crucial for combating latent tuberculosis.

## Data Availability

The datasets generated for this study can be found in NCBI Sequence Read Archive (SRA) database (Accession No. SRR8816592, SRR8816593, SRR8816588, SRR8816589, SRR8816590, SRR8816591, SRR8816605, SRR8816602, SRR8816594, SRR8816595, SRR8816596, SRR8816600, SRR8816603, SRR8816597, SRR8816601, SRR8816604, SRR8816598, SRR8816599).

## Author Contributions

TA, AK, and ES conceived and designed the experiments and wrote the manuscript. ES, AG, OB, YS, and IM performed the experiments. AK, TA, ES, and AG analyzed the data. ES, TA, and AG prepared figures and graphs. All the authors read and approved the final manuscript.

### Conflict of Interest Statement

The authors declare that the research was conducted in the absence of any commercial or financial relationships that could be construed as a potential conflict of interest.

## Supplementary Material

Table S1 Oligonucleotides used in the study.

Table S2 NGS data.

Table S3 Confirmation of NGS data by qPCR. Two different sets of genes were checked for changes in transcripts level in days 2, 4, and 7 vs. dormancy (1), and days 1, 2 vs. dormancy (2). Spearman correlation coefficients between NGS and PCR data were calculated.

Figure S1 Resuscitation of dormant non-culturable *M. tuberculosis* bacilli in the presence of rifampicin. Changes in cell characteristics in the absence (in black) and in the presence (in red) of 5 μg/ml of rifampicin: colony forming units (circles), CFU/ml; radioactive incorporation of uracil (triangles) cpm/ml; optical density (diamonds) OD_600_. This experiment was repeated five times with similar results.

Figure S2 Expression of several protein-coding genes **(A)** and ncRNAs **(B)** at early stages of resuscitation in bacterial cultures with and without rifampicin (rif) treatment. qPCR data are normalized to 16S rRNA transcription level.
