# Supplementary figures and images for: Resuscitation of Dormant “Non-culturable” Mycobacterium tuberculosis Is Characterized by Immediate Transcriptional Burst
Source: Front Cell Infect Microbiol. 2019 Jul 30;9:272. doi: 10.3389/fcimb.2019.00272 (PMC6689984; doi:10.3389/fcimb.2019.00272)

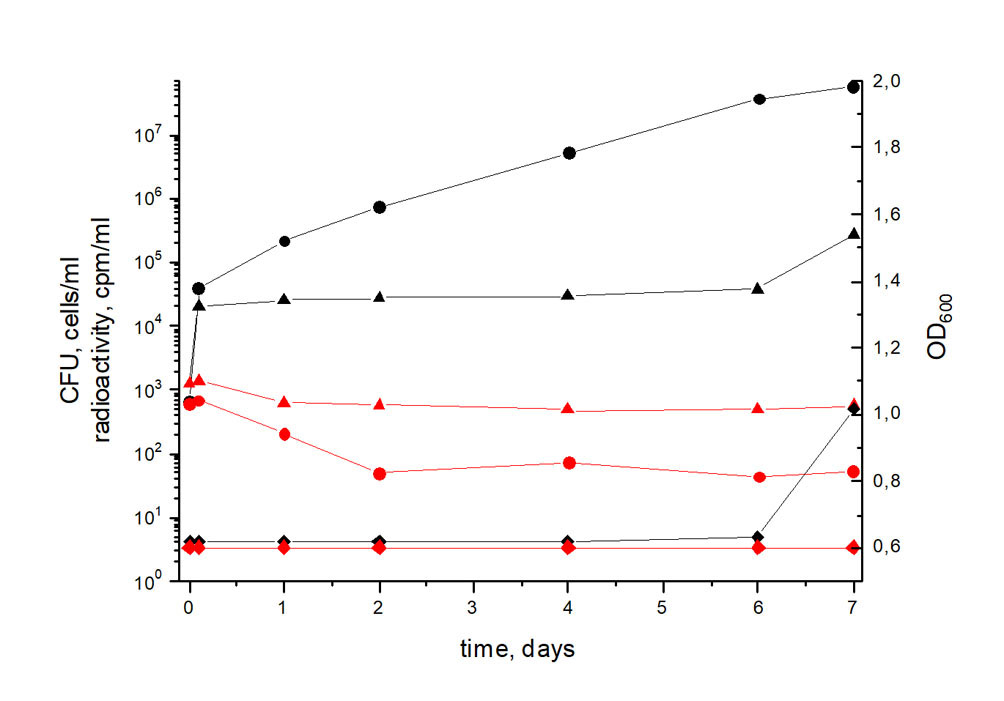

Supplement: Figure S1 — Resuscitation of dormant non-culturable M. tuberculosis bacilli in the presence of rifampicin. Changes in cell characteristics in the absence (in black) and in the presence (in red) of 5 μg/ml of rifampicin: colony forming units (circles), CFU/ml; radioactive incorporation of uracil (triangles) cpm/ml; optical density (diamonds) OD600. This experiment was repeated five times with similar results. [file Image_1.JPEG]

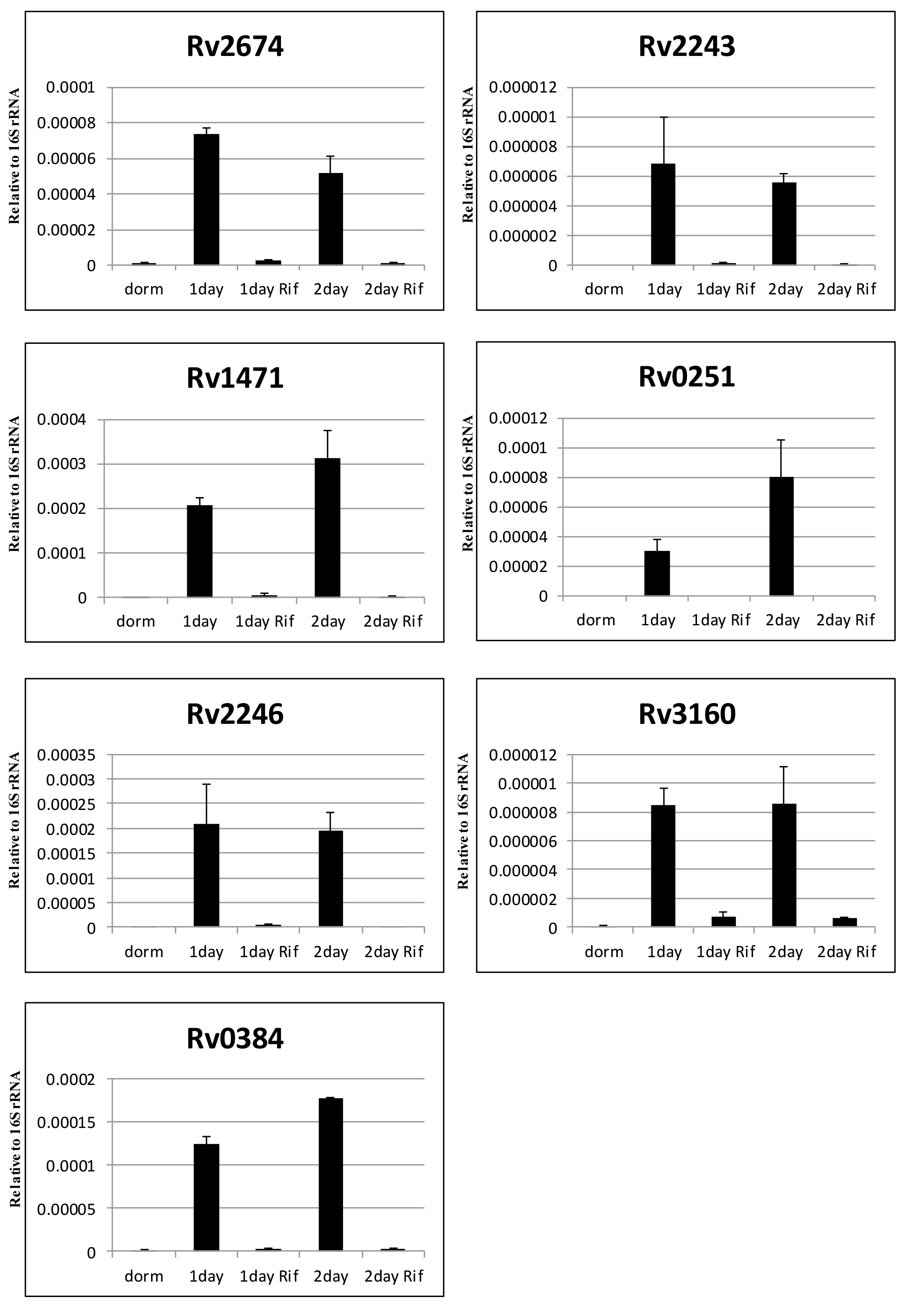

Supplement: Figure S2 — Expression of several protein-coding genes (A) and ncRNAs (B) at early stages of resuscitation in bacterial cultures with and without rifampicin (rif) treatment. qPCR data are normalized to 16S rRNA transcription level. [file Image_2.JPEG]

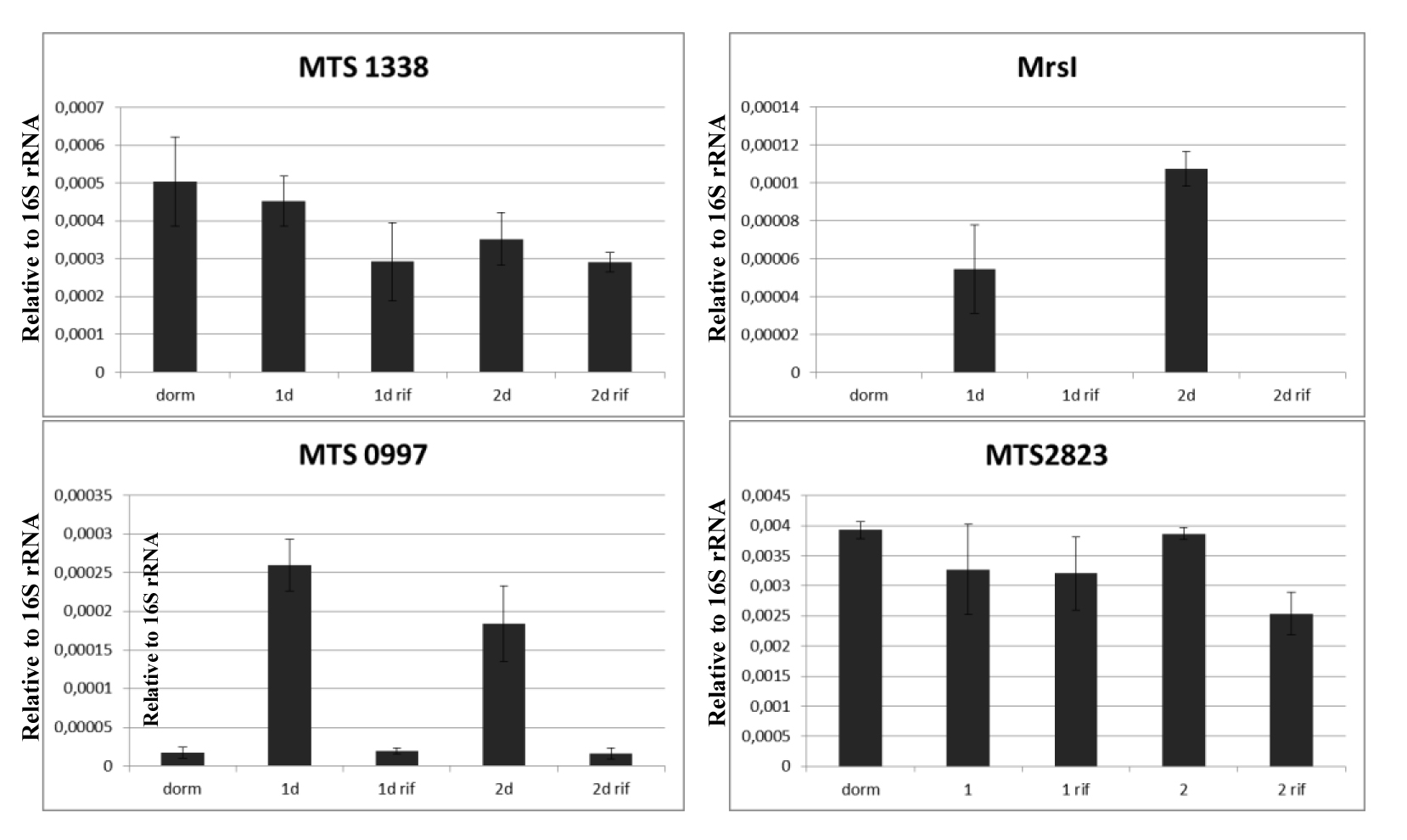

Supplement: Supplementary file 6 [file Image_3.JPEG]
